# Methods of postoperative void trial management after urogynecologic surgery: a systematic review and meta-analysis

**DOI:** 10.1186/s13643-023-02233-1

**Published:** 2023-07-07

**Authors:** Xue Dong, Wu Huang, Jinyang Niu, Tingting Lei, Xin Tan, Tao Guo

**Affiliations:** 1 Ambulatory Surgery Department, West China Second Hospital, Sichuan University, Chengdu, 610041 Sichuan China; 2 Gynecology and Obstetrics Department, West China Second Hospital, Sichuan University, Chengdu, 610041 Sichuan China; 3 Key Laboratory of Birth Defects and Related Diseases of Women and Children (Sichuan University), Ministry of Education, Chengdu, 610041 Sichuan China; 4 Gynecology and Obstetrics Department, People’s Hospital of Pidu District, Chengdu, 611730 Sichuan China; 5 Gynecology and Obstetrics Department, Panzhihua Central Hospital, Panzhihua, 617000 Sichuan China; 6 Gynecology and Obstetrics Department, Suining Municipal Hospital of Traditional Chinese Medical, Suining, 629000 Sichuan China

**Keywords:** Void trial (VT), Force of stream (FOS), Standard voiding trial (SVT), Urogynecologic surgery, Systematic review

## Abstract

**Background:**

Voiding trials are used to identify women at risk for postoperative urinary retention while performing optimal voiding trial management with minimal burden to the patient and medical service team. We performed a systematic review and meta-analysis of postoperative void trials following urogynecologic surgery to investigate (1) the optimal postoperative void trial methodology and (2) the optimal criteria for assessing void trial.

**Method:**

We searched PubMed, EMBASE, Cochrane Central Register of Controlled Trials, and relevant reference lists of eligible articles from inception to April 2022. We identified any randomized controlled trials (RCTs) in English that studied void trials in patients undergoing urogynecologic surgery. Study selection (title/abstract and full text), data extraction, and risk of bias assessment were conducted by two independent reviewers. Extracted study outcomes included the following: the correct passing rate, time to discharge, discharge rate without a catheter after the initial void trial, postoperative urinary tract infection, and patient satisfaction.

**Results:**

Void trial methodology included backfill-assisted and autofill studies (2 RCTs, *n* = 95). Backfill assistance was more likely to be successful than autofill (RR 2.12, 95% CI 1.29, 3.47, *P* = 0.00); however, no significant difference was found in the time to discharge (WMDs =  − 29.11 min, 95% CI − 57.45, 1.23, *P* = 0.06). The criteria for passing void trial included subjective assessment of the urinary force of stream and objective assessment of the standard voiding trial (3 RCTs, *n* = 377). No significant differences were found in the correct passing rate (RR 0.97, 95% CI 0.93, 1.01, *P* = 0.14) or void trial failure rate (RR 0.78, 95% CI 0.52, 1.18, *P* = 0.24). Moreover, no significant differences were found in the complication rates or patient satisfaction between the two criteria.

**Conclusion:**

Bladder backfilling was associated with a lower rate of catheter discharge after urogynecologic surgery. The subjective assessment of FOS is a reliable and safe method for assessing postoperative voiding because it is less invasive.

**Systematic review registration:**

PROSPERO CRD42022313397

**Supplementary Information:**

The online version contains supplementary material available at 10.1186/s13643-023-02233-1.

## Introduction

The reported lifetime risk of urogynecologic procedures is 20% [1–3], and indwelling catheterization is routinely performed. Void trials (VTs) are conducted regularly to guide the decision to discharge patients safely without a catheter [4]. The rates of discharge with a urethral catheter range from 12 to 83% [5], owing to the lack of consensus on the void trial (VT) method and different criteria for passing VT. Elkadry et al. evaluated how patients perceive surgical outcomes and found that 9% believed that being discharged with a catheter was a surgical complication, and 15% named “catheter” as the worst aspect of their surgery [6].

VT usually involves bladder backfill and autofill [7]. Bladder autofill VT involves immediate removal of the Foley catheter postoperatively with a subsequent gradual spontaneous filling of the bladder until a desire to void. The bladder backfill VT is filled retrogradely with a predefined volume of saline (150–300 ml) before the removal of the catheter. The common criteria for assessing VT include subjective assessment of the force of stream (FOS) and objective assessment of the standard voiding trial (SVT). SVT usually involves measuring voided volume (VV) ≥ 68%, and whether or not to check a postvoid residual (PVR) volume < 100 mL, as a definition of a successful VT that patients should be discharged home without a catheter [8]. Ingber et al. published that patient’s subjective reporting of FOS via the visual analog scale (VAS) is safe and efficient to minimize the length of stay and catheter placement for postoperative voiding after mid-urethral sling [9]. They concluded that those with a FOS ≥ 50% were immediately discharged home regardless of PVR urine volume. The strategy for postoperative VT management remains under debate.

Optimal VT management that accurately identifies voiding dysfunction and decreases catheter allocation at discharge is fundamental in providing excellent quality care that may not only reduce overtreatment and overuse of catheters but also expedite recovery. Therefore, a systematic review and meta-analysis of randomized controlled trials (RCTs) were performed to explore the best protocol for managing postoperative voiding trials in patients undergoing urogynecologic surgery.

## Materials and methods

We conducted this review guided by the methods described in the Cochrane Handbook and reported the review using the Preferred Reporting Items for Systematic Reviews and Meta-Analyses (PRISMA) Checklist [10, 11]. This systematic review was registered in the PROSPERO International Prospective Register of Systematic Reviews (https://www.crd.york.ac.uk/PROSPERO/Registration number: CRD42022313397).

### Eligibility criteria

We will include RCTs reporting outcomes after undergoing urogynecologic surgery procedures. Details of the eligibility criteria are as follows: (1) patients—inclusion of patients undergoing urogynecologic surgery procedures; (2) intervention and comparison—comparison of patients undergoing two techniques of VT (bladder backfill VT versus bladder autofill VT) or two criteria for assessing VT (FOS versus SVT); (3) outcomes—the correct passing rate, time to discharge (measured in hours), discharge rate without a catheter after the initial VT, postoperative urinary tract infection (UTI), and patient satisfaction (any kind of reporting). Correct passing was defined as patients discharged after the initial VT without a catheter and not needing to be re-catheterized because of voiding dysfunction. The outcome of UTI was measured based on treatment informed by either lab or clinical symptoms.

Studies that included only one method of VT without a control group, studies that reported duplicated results, those that did not conduct urogynecologic surgery procedures, those including women undergoing pre-operative treatment for urinary retention, and women with neurologic or spinal cord injury affecting bladder function were excluded. Reviews, guidelines, abstracts (insufficient information and data), case reports, conference presentations, editorials, and expert opinions were also excluded.

### Search strategy

Searches were performed using PubMed, EMBASE, and the Cochrane Central Register of Controlled Trials for relevant studies published in English from the dates of inception to April 2022. The electronic search algorithm was designed using Medical Subject Headings (MeSH) terms and keywords for urogynecologic surgery and voiding trials. Details of the complete search strategy are presented in Supplementary Table S1. The reference lists of the included studies were manually searched for potentially relevant studies that were not captured by the electronic search. The search fields “title,” “abstract,” and “MeSH” were applied to ensure the best possible study retrieval. Two researchers (X.D. and W.H.) independently screened to assess the eligibility of the studies. After the initial selection, the full texts of all potential articles were independently read by two researchers (X.D. and W.H.) for further evaluation. Disagreements between authors were resolved by discussion with the T.G.

### Data collection and critical appraisal

Data were independently extracted from the included studies by two researchers (X.D. and W.H.) and were recorded in a standardized sheet. We examined all identified articles that met the inclusion and exclusion criteria of our study. The risk of bias for RCTs was independently assessed by using the Cochrane Risk of Bias Tool. The risk of bias assessment was completed in Review Manager version 5.4. Two reviewers (X.D. and X.T.) independently assessed the quality of the included studies. Discrepancies between reviews were resolved by discussion and consensus with a correspondence (X.T. or T.G.). The risk of bias was analyzed in seven domains: selection bias (random sequence generation allocation concealment), performance bias (blinding of participants and personnel), detection bias (blinding of outcome assessment), attrition bias (incomplete outcome data), reporting bias (selective reporting), and other biases (baseline balance among different groups, no financial support, and effective sample size estimation).

### Statistical analysis

Categorical variables were reported as risk ratios (RRs) with 95% confidence intervals (CIs). Continuous outcomes were reported as weighted mean differences (WMDs) with 95% CIs. Studies expressed their results in median, and interquartile ranges were transformed into mean and standard deviation, using the method described by Hozo and Wan [12, 13]. Heterogeneity among the included studies was assessed using the *I*^2^ statistic. When *I*^2^ was between 0 and 25%, the heterogeneity was considered low, 25–75% was considered moderate, and *I*^2^ > 75% was considered high. When heterogeneity was considered moderate to high, we used random-effect models. Otherwise, a fixed effects model was used. To determine whether the pooled effect was robust, we conducted a sensitivity analysis by omitting one trial at a time and recalculating the summary estimate. When > 10 studies were included, funnel plots were created to determine the possibility of publication bias. All *P-*values were two-sided, and the statistical significance level was set at *P* < 0.05. All statistical analyses were conducted using the RevMan 5.4 software.

## Results

A total of 231 articles were identified using electronic databases. The study selection process is summarized in Supplementary Fig. S1. A total of 162 articles were retrieved after the duplicates were removed. After screening the titles and abstracts, 31 full texts were retrieved for later assessment. Then, 26 articles were excluded after reading the full texts. Finally, five studies met the eligibility criteria. The included studies were then divided into two categories: VT methodology (two studies) [14, 15] and criteria for assessing VT (three studies) [16–18] (Supplementary Table S1). The relevant literature is relatively lacking and involves a small sample size. The majority of studies (four studies) were conducted in the USA, with a single study comparing two different VT methods from Australia. The predominant characteristics of the included studies are summarized in Table 1.

**Table 1:** Characteristics of included studies

Author and year	Setting	Study design	Timing VT	VT method	Intervention (number)	Comparison (number)	VT pass	Outcomes
Trials comparing VT methods								
Mowat, 2018 [15]	Australia	RCT	POD0	Backfill vs. autofill	200 mL backfill (20)	Autofill (20)	PVR < 150 mL with voided volume ≥ 68%	Successful VT rate (95% vs. 90%; *P* = 1); time to discharge (16 h vs. 18.5 h; *P* = 0.43)
Foster, 2007 [14]	USA	RCT	POD0	Backfill vs. autofill	300 mL backfill (27)	Autofill (28)	Voided volume ≥ 200 mL with PVR < voided volume	Successful VT rate (61.5% vs. 32.1%; *P* = 0.02); patient satisfaction (91.7% vs. 96.3%; *P* = 0.22); time to discharge (199.5 min vs. 226.6 min; *P* = 0.08)
Trials comparing VT methods								
Tunitsky-Bitton, 2015 [18]	USA	RCT	POD0	300 mL backfill	FOS (50)	SVT (52)	Int: FOS ≥ 50%; control: voided volume ≥ 200 mL or PVR ≤ 1/3 total volume	Discharge with catheter rate (26.0% vs. 25.5%; *P* = 0.95); void volume (200 vs. 275; *P* = 0.16); PVR (107.5 vs. 66; *P* = 0.4); patient satisfaction (*P* > 0.05); correct passing rate (0/76; 95% CI, 0–5.8%); UTI (6% vs. 5.7%)
Williams, 2019 [16]	USA	RCT	POD0	Backfill	FOS (49)	SVT (53)	Int: FOS ≥ 50% or PVR < 500 mL; control: voided volume ≥ 2/3 total instilled	Discharge without catheter rate (8.2% vs. 9.4%; difference—− 1.3%; 95% CI, − 11.1 to 8.5%; *P* = 1); unexpected postoperative visits rate (10.2% vs. 3.8%; difference 6.0%; 95% CI, − 5.2 to 17.2%); correct passing rate (6.1% vs. 0; difference 6.1% (− 4.0, 16.2)); UTI (4.1% vs. 3.8%; difference 0.3%; 95% CI, − 9.4% to 10.1%); time to discharge (2.1 h vs. 3.2 h; *P* = 0.0002)
Pilkinton, 2019 [17]	USA	RCT	POD0	300 mL backfill	FOS (86)	SVT (87)	Int: FOS ≥ 50%; control: voided volume ≥ 200 mL or 2/3 instilled	Discharge without catheter rate (17.4% vs. 26.4; RR 0.65, 95% CI 0.37–1.18, *P* > 0.05); correct passing rate (2.8% vs. 3.1%; difference 20.31% 95% CI 28.69 to 8.08%); UTI (9% vs. 7%; *P* > 0.05)

*VT* Void trial, *Int* Interventional group, *RCT* Randomized controlled trial, *PACU* Post-anesthesia care unit, *UTI* Urinary tract infection, *POD* Postoperative day, *FOS* Force of stream, *SVT* Standard voiding trial, *PVR* Postvoid residual, *CI* Confidence interval, *RR* Risk ratio

### Quality of the studies

**Fig. 1:**
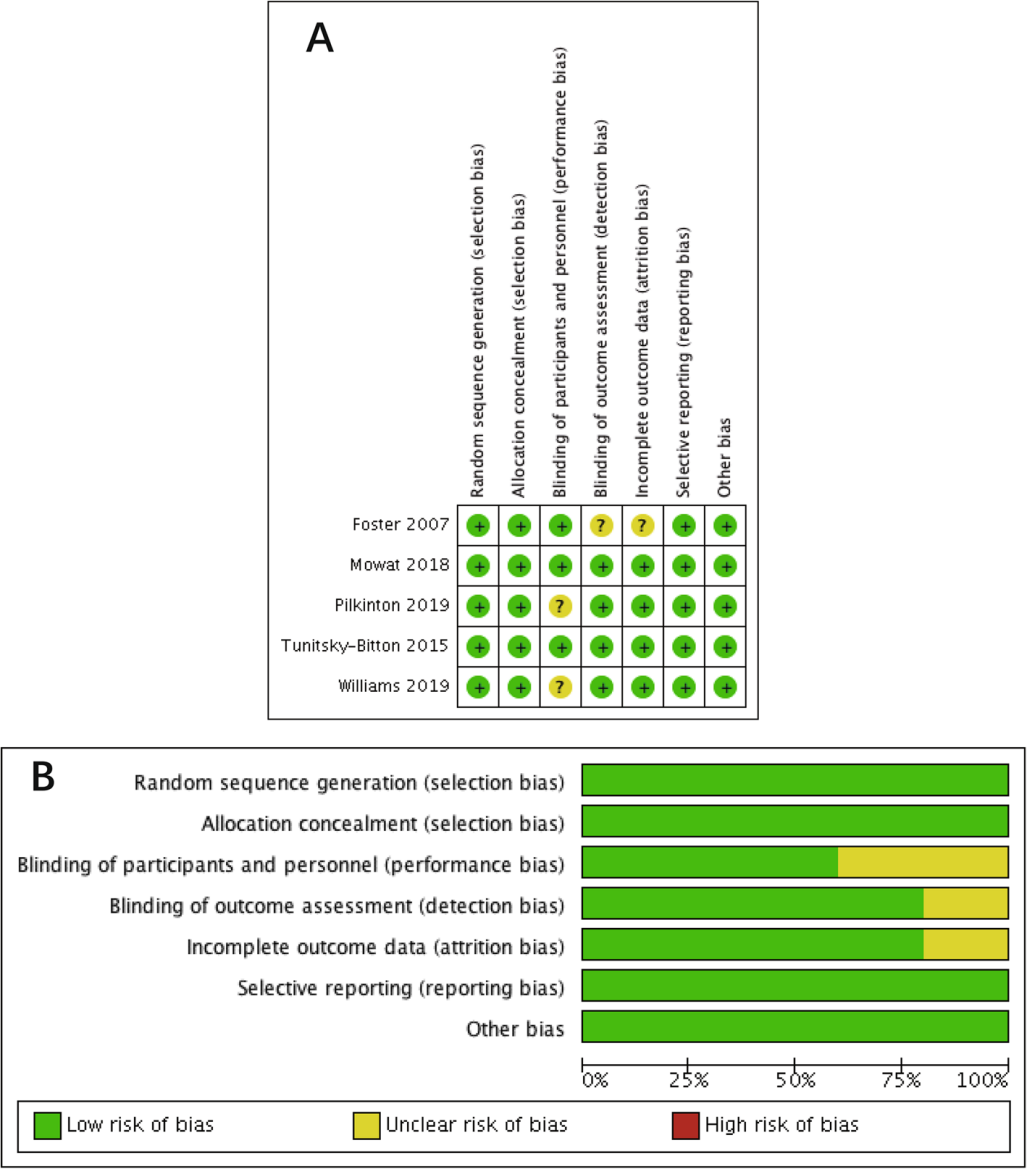
Risk of bias assessment, including graph (**A**) and summary (**B**)

The Preferred Reporting Items for Systematic Reviews and Meta-Analyses (PRISMA) flowchart is shown in Supplementary Table S2. The summary of the risk of bias assessment is shown in Fig. 1. All studies were randomized. Two studies did not carefully describe the blinding technique used. Foster et al. reported an unclear risk of attrition bias. No other source of bias was identified. Overall, all studies were assessed as having a low risk of bias.

### Trials comparing void trial methods

**Fig. 2:**
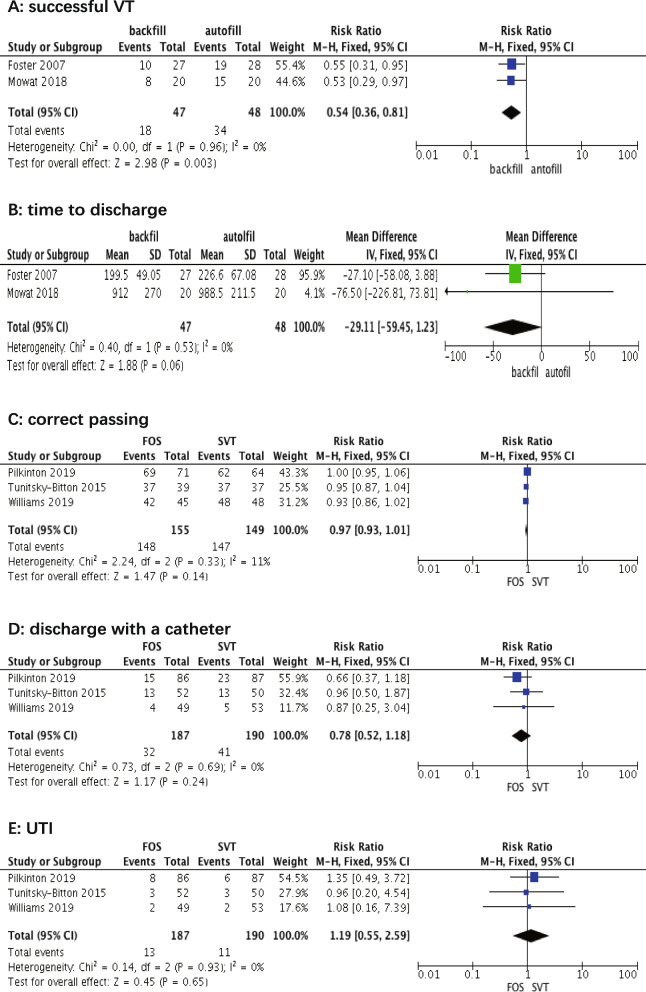
Meta-analysis considering trials comparing VT methods (**A** successful VT, **B** time to discharge) and trials comparing criteria for passing VT (**C** correct passing, **D** discharge with a catheter, **E** UTI) according to the forest plot

Two RCTs with a total of 95 participants were included. All studies were assessed as having a low risk of bias. VT methodology included backfill-assisted and autofill studies. The definition of successful VT varied across studies (Table 1), with a combination of VV and PVR. The time to discharge was the primary outcome in both studies. A successful VT is defined as a discharge without a catheter. For the primary endpoint, VT was significantly more likely to be successful in the bladder backfill-assisted group than in the autofill VT (RR 2.12, 95% CI 1.29 to 3.47, *P* = 0.00, *I*^2^ = 0%; fixed effects model) (Fig. 2). The time to discharge did not differ significantly between the two groups (mean difference − 29.11 min; 95% CI − 57.45 to 1.23, *P* = 0.06, *I*^2^ = 0%, fixed effects model) (Fig. 2). Only one included study measured the satisfaction survey by questionnaires. Foster et al. found no difference in the proportion of subjects satisfied with the VT technique between those who had a backfill-assisted VT and those who had a spontaneous VT.

### Trials comparing criteria for assessing VT

Three RCTs with a total of 377 participants were included. All studies were assessed as having a low risk of bias. The criteria for assessing VT included the FOS and SVT. All studies used the backfill-assisted VT method. Our outcomes included the correct passing rate after the initial VT assessment, the rate of discharge with a catheter, postoperative UTI, and patient satisfaction. There was no significant difference between the two criteria (RR 0.97, 95% CI 0.93 to 1.01, *P* = 0.14, *I*^2^ = 11%, fixed effects model) (Fig. 2). For patients who failed VT and required re-catheterization at discharge, no significant difference was observed between the two groups (RR 0.78, 95% CI 0.52–1.18, *P* = 0.24, *I*^2^ = 0%, fixed effects model) (Fig. 2). Finally, we observed that the pooled UTI outcomes did not show a significant difference (RR 1.19, 95% CI 0.55–2.59), with *P* = 0.65 and heterogeneity *I*^2^ = 0% (Fig. 2). Only Tunitsky-Bitton et al. reported patient satisfaction by questionnaires, and there were no significant differences between the two criteria (*P* > 0.05).

## Discussion

Our current study found that bladder backfill-assisted VT is associated with a lower rate of discharge with a catheter; however, it does not translate to an earlier discharge from the hospital in the meta-analysis. We also observed no statistically significant difference in the correct passing rate, discharge with a catheter rate, or UTI between FOS and SVT for the assessment of postoperative voiding. Additionally, we found no statistically significant impact on postoperative patient satisfaction.

A recent systematic review by Dieter et al. showed that VT methodologies using backfill-assisted, autofill, and FOS resulted in similar outcomes, with no one method being superior after gynecologic and urogynecologic surgery [19]. However, that review regarded FOS as a postoperative VT methodology and presented it in tabular form. Based on prior studies [9, 16–18, 20, 21], we believe that it is probably more appropriate to consider the FOS as a method of postoperative VT assessment. In addition, our review was specific to urogynecologic procedures and analyzed the clinical feasibility and safety of postoperative VT management, not just the VT method, but also the criteria for assessing VT. To the best of our knowledge, this is the first systematic review to focus on postoperative VT in urogynecologic surgery patients, including the optimal postoperative VT methodology and the optimal criteria for assessing VT.

Currently, the backfill VT has been shown to have greater accuracy [22] and cost savings than the autofill method [23]. When comparing the time to discharge, we did not find evidence to support the superiority of backfill assistance. This is consistent with our previous study on two VT methods after outpatient laparoscopic gynecological surgery [24]. However, backfill-assisted surgery was associated with a higher rate of successful VT. To our knowledge, higher VT success results in potentially fewer catheters assigned at discharge and less affected quality of life because living with an indwelling catheter accompanies discomfort and difficulties [25]. Considering the lack of studies on postoperative complications, including UTI and readmission rate, it is impossible to compare the safety of the two VT methods.

Traditionally, objective indicators of VT, including VV and PVR, are considered mandatory before discharge [26, 27]. However, these strategies have several limitations. Assessing PVR requires a bladder scan or an ultrasound machine with special skills, which may not always be readily available [28]. Previous studies have also shown that even low-cost routine interventions are responsible for substantial healthcare expenditures [29]. Postoperative urinary retention was similar among those with a strict voiding protocol and those discharged with their subjective determination after gynecologic and urogynecologic surgery [30], with a negative predictive value of 97% [31]. Our results show that the less stringent criteria used in the FOS protocol are safe and effective and do not appear to increase the risk of catheterization and voiding dysfunction after discharge. Everything has two aspects: The FOS approach requires additional patient and nurse training and allows for potential patient bias [32]. Patients may be motivated to report an increased FOS, knowing that their assessment will influence the decision to be discharged with or without a catheter.

The strength of our review is the thorough literature research. We also evaluated the risk of bias using the Cochrane Collaboration tool. Moreover, a wide variety of surgical approaches were involved, including both suspension and obliterative procedures, with and without concomitant hysterectomy or sling. Different urogynecology procedures may have impacted the efficacy or feasibility of VT. For example, prolapse repair likely increases the need for catheterization due to the change in configuration of the bladder and vaginal tissue, increased edema, increased anesthesia time, and increased opioid use. Considering the abovementioned studies, the criteria for assessing VT had similar baseline participant characteristics in both groups. Thus, our findings may be generalized and used in urogynecology as well as gynecological practices performing these procedures.

Our study has some limitations. Literature is relatively absent, and a significant amount of heterogeneity appears in the included studies due to the differences in the quality of evidence, details of VT protocols, and patient populations. Although some studies were at risk of bias due to incomplete blinding of the participants and outcome assessors, they all designed an objective way to measure the outcomes. Furthermore, ward nursing staff, the culture of the hospital system, and pre-operative expectations may represent sources of potential bias influencing the outcomes. Subsequently, we attempted to explore this heterogeneity by gaining a better understanding of the nuances of the interventions. The included studies of VT methods did not have a clear baseline participant characteristic in either group, especially the performance of incontinence procedures, the use of mesh, or the need for apical suspensions, which may impact the likelihood of success of the voiding trial [33, 34]. We used a one-by-one elimination method to determine the presence of clinical or methodological heterogeneity. All of these efforts failed. In addition, the population was mostly from the USA, with only one study conducted in Australia, which may limit the generalizability of the results and their applicability to more racially diverse populations elsewhere.

We suppose that these results should be interpreted with caution based on the relative lack of literature on the topic after urogynecologic surgery and the limited statistical power to detect a difference in these infrequent outcomes. Larger and appropriately powered data collection would be required to validate our analysis. Because less than 10 studies were included in the meta-analysis, funnel plot asymmetry was not taken into account.

## Conclusion

In summary, bladder backfilling is associated with a lower rate of catheter discharge after urogynecologic surgery. In contrast, the two criteria for assessing VT seem efficacious and safe for evaluating postoperative voiding. The subjective assessment of FOS is also less invasive, more reliable, and does not require additional interventions to measure PVR. More large trials with appropriate cost-effectiveness analyses are needed to standardize recommendations and demonstrate for sure that both individual patients and health systems would benefit.

## Supplementary Information

**Additional file 1.** PRISMA Checklist.

**Additional file 2.** Search strategy.

**Additional file 3: Fig. S1.** Flow diagram demonstrating the study selection process.

## Data Availability

All data generated or analyzed during this study are included in this published article [and its supplementary information files].
