# Supplementary material for: Methods of postoperative void trial management after urogynecologic surgery: a systematic review and meta-analysis
Source: Syst Rev. 2023 Jul 7;12:115. doi: 10.1186/s13643-023-02233-1 (PMC10327332; doi:10.1186/s13643-023-02233-1)
Supplement: Supplementary file 2 — Additional file 2. Search strategy. [file 13643_2023_2233_MOESM2_ESM.docx]

**Supplementary Table 1:** Search strategy for present systematic review and meta-analysis

**PubMed search**

| **Number** | **Search** | **Results** |
| --- | --- | --- |
| 1 | **“****Urogynecologic Surgical Procedures’’[Mesh] OR Suburethral Slings’’[Mesh] OR “Urinary Incontinence/surgery’’[Mesh] OR “incontinence surgery”[tiab] OR “incontinence surgeries”[tiab] OR “anti-incontinence surgery”[tiab] OR “anti-incontinence surgeries”[tiab] OR “Suburethral Slings’’[tiab] OR “Suburethral Sling’’[tiab] OR “midurethral slings”[tiab] OR “midurethral sling”[tiab]** | 11411 |
| **2** | **“Pelvis’’[Mesh] OR “Prolapse’’[Mesh] OR urogynecologic[tiab] OR urogynecological[tiab] OR urogynecology[tiab] OR pelvic[tiab] OR pelvis[tiab] OR prolapse[tiab] OR prolapses[tiab] OR uterus[tiab] OR vagina[tiab]** | 267806 |
| 3 | **“Surgical Procedures, Operative’’[Mesh] OR “surgery’’[Subheading] OR “General Surgery’’[Mesh] OR operative[tiab] OR operation[tiab] OR operations[tiab] OR surgery[tiab] OR surgeries [tiab] OR surgical[tiab] OR surgeon[tiab] OR surgeons[tiab] OR procedure[tiab] OR procedures [tiab] OR elective[tiab] OR reconstruction[tiab] OR reconstructions[tiab] OR repair[tiab] OR repairs[tiab]** | 5983423 |
| 4 | **"retrofill"[All Fields] OR "retrofilled"[All Fields] OR "retrofilling"[All Fields] OR "retrofillings"[All Fields] OR "retrofills"[All Fields] or "retrograde"[All Fields] OR "retrogradely"[All Fields] or "backfill"[All Fields] OR "backfilled"[All Fields] OR "backfilling"[All Fields] OR "backfills"[All Fields] or "infusate"[All Fields] OR "infusates"[All Fields] OR "infuse"[All Fields] OR "infused"[All Fields] OR "infuser"[All Fields] OR "infusers"[All Fields] OR "infuses"[All Fields] OR "infusing"[All Fields] OR "infusion"[All Fields] OR "infusions"[All Fields] or (("catheter's"[All Fields] OR "catheters"[MeSH Terms] OR "catheters"[All Fields] OR "catheter"[All Fields]) and "catheter's"[All Fields] OR "catheters"[MeSH Terms] OR "catheters"[All Fields] OR "catheter"[All Fields])** | 604431 |
| **5** | **(void or voiding or post void or post-void or postvoid).af.** | 53212 |
| 6 | **(#2 and #3) or #1** | 3906 |
| 7 | **(#4 or #5) and #6** | 676 |
| 8 | **(randomized controlled trial[pt] OR controlled clinical trial[pt] OR randomized[tiab] OR placebo [tiab] OR drug therapy[sh] OR randomly[tiab] OR trial[tiab] OR groups[tiab] NOT (animals [mh] NOT humans [mh]))** | 4734505 |
| 9 | **#7 and #8** | 223 |

**Embase search**

| **Number** | **Search** | **Results** |
| --- | --- | --- |
| **1** | **(Urogynecologic surgery or urologic surgery or suburethral sling or incontinence surgery or incontinence surgeries or anti-incontinence surgery or antiincontinence surgeries or Suburethral Slings or Suburethral Sling or midurethral slings or midurethral sling).af.** | **16217** |
| **2** | **(pelvis or prolapse or urogynecologic or urogynecological or urogynecology or pelvic or prolapse or prolapses or vagina).af.** | **268916** |
| **3** | **(surgery or operative or operation or operations or surgeries or surgical or surgeon or surgeons or procedure or procedures or reconstruction or reconstructions or repair or repairs).af.** | **5600630** |
| **4** | **(retrofill or retrofilled or retrofilling or retrofillings or retrofills or retrograde or retrogradely or backfill or backfilled or backfilling or backfills or infusate or infusates or infuse or infused or infuser or infusers or infuses or infusing or infusion or infusions).af or (catheter's or catheters or catheter).af.** | **642526** |
| **5** | **void or voiding or post void or post-void or postvoid** | **30223** |
| 6 | **(#2 and #3) or #1** | **165393** |
| 7 | **(#4 or #5) and #6** | **13913** |
| 8 | **limit 40 to (female and english language and randomized controlled trial)** | **312** |

**Cochrane Central Register of Controlled Trials search**

| **Number** | **Search** | **Results** |
| --- | --- | --- |
| **1** | **[mh“Urogynecologic surgery Procedures”] or [mh“Urologic Surgical Procedures”] or [mh’’Urinary Incontinence”] or [mh“Suburethral Slings”] or incontinence surgery ti,ab or incontinence surgeries ti,ab or Suburethral Slings ti,ab or Suburethral Sling ti, ab or midurethral slings ti,ab or midurethral sling ti,ab** | **9837** |
| **2** | **[mh ’’Gynecology’’] or [mh ’’Pelvis’’] or [mh ’’Genital Diseases, Female’’] OR [mh ’’Prolapse’’] or [mh ’’Genitalia, Female’’] or urogynecologic ti, ab or urogynecological ti,ab or urogynecology ti,ab or pelvic:ti,ab or pelvis:ti,ab or prolapse ti,ab or prolapses ti,ab or vagina:ti,ab** | **23905** |
| **3** | **[mh ’’Surgical Procedures, Operative’’] or [mh ’’General Surgery’’] or operative ti,ab or operation ti,ab or operations ti,ab or surgery ti,ab or surgeries ti,ab or surgical ti,ab or surgeon ti,ab or surgeons ti,ab or procedure ti,ab or procedures:ti,ab or reconstruction ti,ab or reconstructions ti,ab or repair ti,ab or repairs:ti,ab** | **151236** |
| **4** | **#2 and #3** | **7004** |
| **5** | **#1 or #4** | **8340** |
| **6** | **[mh ’’Postoperative Period’’] or postoperative ti,ab or post-operative ti, ab or post-operatively ti,ab or post-anesthesia ti,ab** | **7761** |
| **7** | **Voiding ti,ab or void ti,ab or postvoid ti,ab or post-void ti,ab or ‘post void’ ti,ab** | **899** |
| **8** | **#5 and #6 and #7** | **28** |

**Study Registry: ClinicalTrials.gov**

| **Number** | **Search** | **Results** |
| --- | --- | --- |
| 1 | **(voiding OR void OR postvoid OR post-void OR ’’post void’’** |  |
| 2 | **(sling OR slings OR hysterectomy OR hysterectomies OR surgery OR surgical OR surgeries OR repair OR repairs OR reconstruction OR reconstructions) AND (postoperative OR post-operative OR ’’post operative’’)** |  |
| 3 | **（#1 AND #2）Filter to Interventional Studies; Completed with Results** | 38 |
