# Supplementary figures and images for: Methods of postoperative void trial management after urogynecologic surgery: a systematic review and meta-analysis
Source: Syst Rev. 2023 Jul 7;12:115. doi: 10.1186/s13643-023-02233-1 (PMC10327332; doi:10.1186/s13643-023-02233-1)

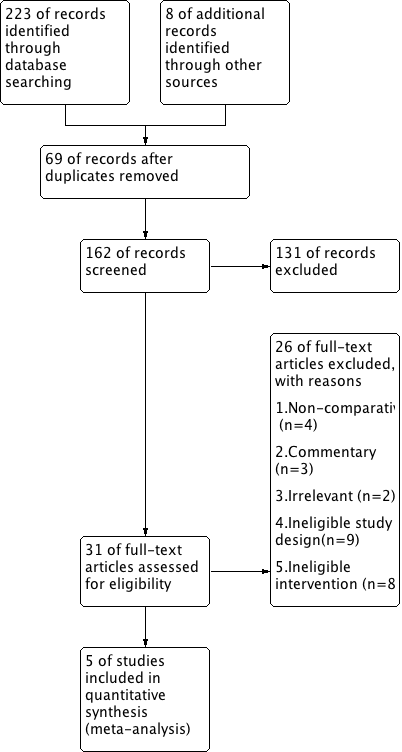


Flow diagram demonstrating the study selection process

Supplement: Supplementary file 3 — Additional file 3: Fig. S1. Flow diagram demonstrating the study selection process. [file 13643_2023_2233_MOESM3_ESM.docx]
